# An Unusual Carbohydrate Conformation is Evident in *Moraxella catarrhalis* Oligosaccharides

**DOI:** 10.3390/molecules200814234

**Published:** 2015-08-05

**Authors:** Martin Frank, Patrick M. Collins, Ian R. Peak, I. Darren Grice, Jennifer C. Wilson

**Affiliations:** 1 Biognos AB, Generatorsgatan 1, 41705 Gothenburg, Sweden; E-Mail: martin@glycosciences.org; 2 Institute for Glycomics, Gold Coast Campus, Griffith University, 4222 Queensland, Australia; E-Mail: patrick.collins@diamond.ac.uk; 3 Institute for Glycomics and School of Medical Science, Gold Coast Campus, Griffith University, 4222 Queensland, Australia; E-Mails: i.peak@griffith.edu.au (I.R.P.); d.grice@griffith.edu.au (I.D.G.); 4 Menzies Health Institute and School of Medical Science, Gold Coast Campus, Griffith University, 4222 Queensland, Australia

**Keywords:** carbohydrate conformations, oligosaccharides, molecular dynamics simulations, NMR

## Abstract

Oligosaccharide structures derived from the lipooligosaccharide of *M. catarrhalis* show that the highly branched glucose-rich inner core of the oligosaccharide has an altered conformation compared to the most truncated tetra-glucose-Kdo lgt1/4Δ oligosaccharide structure. Addition of one residue each to the (1-4) and (1-6) chains to give the lgt2Δ oligosaccharide is the minimum requirement for this conformational change to occur. Extensive molecular modeling and NMR investigations have shown that the (1-3), (1-4), and (1-6) glycosidic linkages from the central α-d-Glc*p* have significantly altered conformational preferences between the two structures. For the lgt1/4Δ oligosaccharide the (1-3) and (1-4) linkage populates predominantly the syn minimum on the conformational free energy map and for the (1-6) linkage conformational flexibility is observed, which is supported by ^1^H-NMR T1 measurements. For the lgt2Δ oligosaccharide the unusual “(1-4)anti-ψ(1-6)*gg*” conformation, which could be confirmed by long-range NOE signals, is a dominant conformation in which the oligosaccharide is very compact with the terminal α-d-GlcNAc residue folding back towards the center of the molecule leading to an extensive intra-molecular hydrophobic interaction between the terminal residues. Comparing effective H-H distances, which were calculated for conformational sub-ensembles, with the NOE distances revealed that typically multiple conformations could be present without significantly violating the measured NOE restraints. For lgt2Δ the presence of more than one conformation is supported by the NOE data.

## 1. Introduction

The pathogenic bacterium *Moraxella catarrhalis* is one of the causes of the most common disease diagnosed amongst children: otitis media (or middle ear infection) [1,2]. The lipooligosaccharide (LOS) component of the outer membrane of *M. catarrhalis* is a unique and complex class of glycolipid that is important in bacterial infection and represents an attractive vaccine target [3,4,5,6]. These amphiphilic molecules consist of a hydrophobic component, termed lipid A, that anchors the molecule into the outer membrane, and a hydrophilic oligosaccharide (OS) component. Although LOS molecules are structurally diverse between bacterial species, the lipid A component generally consists of a phosphorylated β-1,6-diglucosamine backbone with a number of *N*- and *O*-linked acyl groups, and is linked to a highly branched OS chain through a 3-deoxy-α-d-manno-oct-2-ulopyranosidic acid (Kdo) molecule [3,7,8].

**Figure 1:**
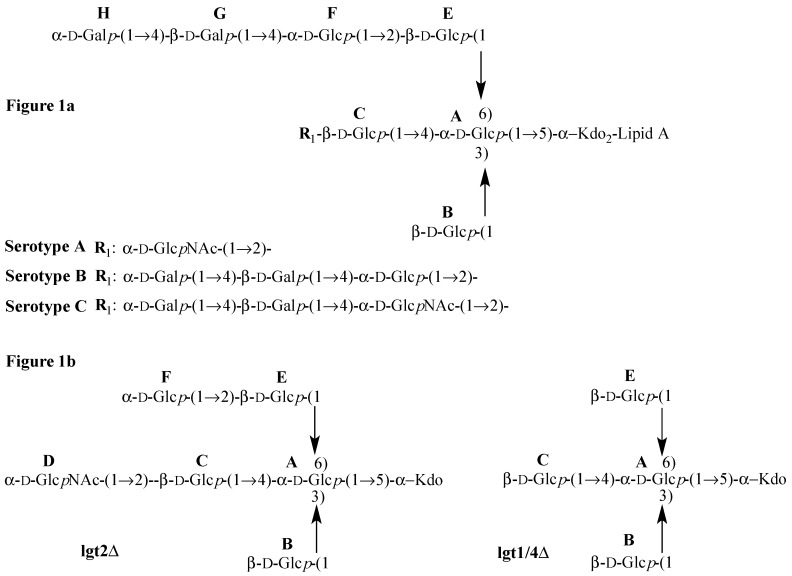
Structures of (**a**) wild-type serotypes **A**, **B** and **C** *Moraxella catarrhalis* LOS (OS highlighted (sugar labeling according to Lycknert *et al*. [20]); and (**b**) serotype **A** lgt2Δ and lgt1/4Δ OS.

Three major serotypes (**A**, **B**, and **C**) account for 95% of clinical isolates of *M. catarrhalis* in one study [9], and it is the OS component of the LOS that is the chemical basis for the antigenic difference between these serotypes [10]. The primary sugar sequence of the OS from each of the major serotypes has been determined [11,12,13] and reveals that all contain a common branched pentasaccharide core (Figure 1 sugars **A**, **B**, **C**, **E**, and **F**) linked through two Kdo residues to lipid A. An unusual feature of this pentasaccharide inner core OS of *M. catarrhalis* is that it does not contain heptose residues and the inner core instead is comprised of glucose residues, one of which is directly bonded to Kdo. Depending on the serotype, up to an additional 2-3 hexose residues may be present on the 1-4 and 1-6 linked chains (Figure 1). Recently, we [14,15] and others [16,17,18,19] have begun to unravel the roles of the various glycosyltransferases that direct the serotype specific biosynthesis of the LOS in *M. catarrhalis*.

Interestingly, Lycknert *et al.* [20] reported that once a critical number of two sugar residues are present on the 1-4 and 1-6 chains (along with residue **B**, giving six hexoses), a significant conformational change occurs in the three-dimensional structure of the OS in *M. catarrhalis*. The occurrence of this conformational change was signaled by significant ^1^H-NMR chemical shift changes for certain residues in isolated and synthesized OS [20]. Furthermore, they observed that the presence of additional sugar residues on either the 1-4 and 1-6 chains yields no further alteration in the chemical shift values.

We have recently characterized a number of OSs isolated from LOS mutant *M. catarrhalis* strains that lack specific glycosyltransferases involved in LOS biosynthesis [15]. These mutants produced truncated OS, and we too have observed notable ^1^H-NMR chemical shift differences for the more highly truncated OS structures compared to wildtype. Herein, we describe the nature of the conformational change that occurs in the OS of *M. catarrhalis* LOS, and the three-dimensional structure of representative OS as determined by NMR and an extensive conformational analysis based on data derived from molecular dynamics (MD) simulation.

## 2. Results and Discussion

**Figure 2:**
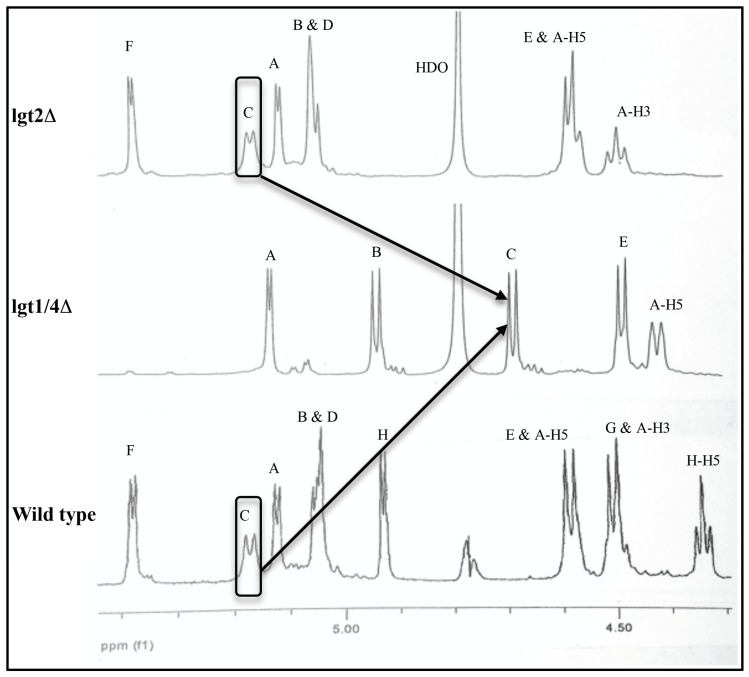
Comparison of the anomeric ^1^H-NMR (ppm, D_2_O, 298 K, 600 MHz) regions of serotype A wild type, lgt2Δ and lgt1/4Δ OS.

The two selected OS, isolated from mutant serotype A *M. catarrhalis* [15], are referred to as lgt2Δ, a heptasaccharide (lacking sugars **G** and **H**, but with an additional sugar labeled **D**) and lgt1/4Δ, a pentasaccharide lacking the terminal sugar residues **D** and **F** (shown in Figure 1b) compared to lgt2Δ. The lgt2Δ OS contains the minimum critical number of two residues on the (1-4) and also the (1-6) linked chains to present a conformation consistent with wildtype OS and therefore it also displays similar ^1^H-NMR chemical shifts to that of wild type OS [12], and other larger OS that are also extended on the (1-6) chain [14]. The presence of only one residue (**C**) on the (1-4) linked chain in lgt1/4Δ results in significantly altered ^1^H-NMR chemical shifts compared to lgt2Δ, especially in the anomeric proton region (4.48–5.41 ppm) (Figure 2, Table 1). The anomeric proton signals of all three β-d-glucopyranose residues (**B**, **C**, and **E**) are shifted downfield (deshielded) in the larger lgt2Δ OS compared to lgt1/4Δ (up to 0.48 ppm for **C**) (see Table 1). This effect is not simply due to chemical substitution at the 2′ positions of residues **C** and **E**, since the anomeric proton signal from the unsubstituted residue **B** is also very significantly altered. Additionally, when residue **F** (on the 1–6 linked chain) is further substituted with one [14] or two [12] additional sugar residues, no further ^1^H-NMR chemical shifts are observed.

**Table 1:** Comparison of ^1^H-NMR data (ppm, D_2_O, 298 K, 600 MHz [15]) for serotype A wild type, lgt2Δ and lgt1/4Δ OS (wild type data from Edebrink [12], on serotype A (25238), while lgt2Δ and lgt1/4Δ data is from Peak *et al.* [15] (serotype A)).

Sugar Residue	Oligosaccharide	H1	H2	H3	H4	H5
**A**	Wild type	5.16	3.90	4.53	3.95	4.62
	lgt2Δ	5.11	3.89	4.49	3.91	4.57
	lgt1/4Δ	5.13	3.80	4.26	3.95	4.41
**B**	Wild type	5.08	3.36	3.55	3.36	3.47
(3-linked to **A**)	lgt2Δ	5.04	3.34	3.51	3.32	3.54
	lgt1/4Δ	4.93	3.36	3.49	3.38	3.43
**C**	Wild type	5.20	3.39	3.60	3.44	3.51
(4-linked to **A**)	lgt2Δ	5.16	3.36	3.57	3.43	3.47
	lgt1/4Δ	4.68	3.33	3.49	3.38	3.44
**D**	Wild type	5.08	4.03	3.74	3.58	3.85
(2-linked branch to **C**)	lgt2Δ	5.05	3.99	3.71	3.55	3.80
	lgt1/4Δ	-	-	-	-	-
**E**	Wild type	4.62	3.49	3.58	3.44	3.44
(6-linked to **A**)	lgt2Δ	4.58	3.46	3.55	3.37	3.41
	lgt1/4Δ	4.48	3.30	3.49	3.38	3.43
**F**	Wild type	5.41	3.45	3.88	3.64	4.12
(2-linked branch to **E**)	lgt2Δ	5.38	3.35	3.73	3.39	3.97
	lgt1/4Δ	-	-	-	-	-

In order to study these effects, further nuclear Overhauser enhancements (NOEs) were measured on the two OS (lgt2Δ and lgt1/4Δ) using a combination of 2D NOESY and 2D HSQC-NOESY NMR experiments. In addition to many intra-residual NOEs, many inter-residual NOEs across the glycosidic linkages were also measured. Inter-residual NOEs provide information about the torsion angles in a glycosidic linkage and can be used to piece together the overall structure of an OS. For the lgt2Δ OS, we also observed many NOEs between further remote residues, such as between protons on residues **F** and **D**, separated by three sugar residues. These types of NOEs are rare in OS due to their generally flexible and non-rigid nature [21], however we acquired measurements of at least eight “long-range” NOEs for lgt2Δ. The presence of so many “long-range” NOEs indicates that the OS from *M. catarrhalis* LOS may be highly constrained in their motion and form very specific structures. The NOEs derived for lgt1/4Δ and lgt2Δ are shown in Table 2 and Table 3, respectively.

There are several fundamental intrinsic problems in deriving a three-dimensional (3D) model of an OS based on NOE intensities: in solution the OS might exist as a mixture of different conformations and each of them contributes to the NOE intensities to some extent. In this case a single local minimum conformation that satisfies all restraints may not exist. Even if such a single model could be built, additional conformations could remain undetected because they are “shadowed” by the major conformation. This could happen for example if they share some of the NOEs with the major conformation and other “reporter” NOEs—which are unique for the additional conformation—are either too weak to be detected or located in a region of the spectrum with signal overlap. In order to support the interpretation of the NMR results one would aim at calculating a representative conformational ensemble using molecular dynamics (MD) simulations at room temperature in explicit solvent and compare ensemble statistics of H-H distances with effective NOE distances calculated from relative NOE signal intensities. Despite significant improvements in the development of carbohydrate force fields and in sampling technology like accelerated molecular dynamics [22], replica-exchange molecular dynamics [23,24,25] and Hamiltonian replica-exchange simulation [26,27,28,29], it is still very difficult to generate efficiently and reliably a converged room-temperature ensemble in explicit solvent that provides a basis for reliable interpretation and also to some extent validation of the NMR data. In the case of a discrepancy between the ensemble averaged *r*_eff_ and the effective NOE distance it will remain unclear whether the measured NOE intensity or the simulation results are in question.

**Table 2:** Inter-residue NOE data obtained for lgt1/4Δ OS. Distance constraints used for classification: strong 1.8–2.8 Å, medium 1.8–3.3 Å and weak 1.8–5.0 Å. The effective H-H distance *r*_NOE_ was calculated from the peak integral *I* using equation *r*_NOE_ = *r_ref_* (*I_ref_*/*I*)^1/6^. * Virtual NOE distance, multiple H-H pairs contribute, ** H6_1_ or H6_2_.

Label	Classification	NOE Distance [Å]
A-H1⇔Kdo-H5	strong	2.4
B-H1⇔A-H3	strong	2.6
C-H1⇔A-H4	strong	2.7
C-H1⇔A-H6_1_/H6_2_	composite	2.6 *
E-H1⇔A-H6_1_/H6_2_	composite	2.4 *
E-H1⇔A-H6_x_	medium	2.9 **
A-H1⇔A-H2	reference	2.46

**Table 3:** Inter-residue NOE data obtained for lgt2Δ OS. Distance constraints used for classification: strong 1.8–2.8 Å, medium 1.8–3.3 Å and weak 1.8–5.0 Å. The effective H-H distance *r*_NOE_ was calculated from the peak integral *I* using equation *r*_NOE_ = *r_ref_* (*I_ref_*/*I*)^1/6^. * Virtual NOE distance, multiple H-H pairs contribute.

Label	Classification	NOE Distance [Å]
A-H1⇔Kdo-H5	strong	2.2
A-H1⇔Kdo-H6	weak	3.4
A-H1⇔Kdo-H7	strong	2.6
A-H1⇔Kdo-H4	weak	3.4
B-H1⇔A-H3	medium	2.9
B-H1⇔C-H2	medium, long	2.9
C-H1⇔A-H3	medium	3.0
C-H1⇔A-H5	medium	3.2
E-H1⇔A-H6_1_/H6_2_	composite	2.6 *
D-H1⇔C-H2	strong	2.6
F-H1⇔E-H1	weak	3.6
F-H1⇔E-H2	strong	2.5
F-H1⇔A-H4	medium, long	2.8
F-H1⇔A-H6_1_/H6_2_	composite, long	3.1 *
F-H2⇔D-H2	weak, long	3.3
D-CH_3_⇔F-H1	composite, long	3.2 *
D-CH_3_⇔E-H1	composite, long	3.5 *
D-CH_3_⇔A-H2	composite, long	3.0 *
D-CH_3_⇔A-H4	composite, long	2.8*
D-CH_3_⇔B-H2	composite, long	2.8 *
A-H1⇔A-H2	reference	2.46

Considering the still-existing practical limitations in the simulation of converged conformational ensembles of carbohydrates in solution, our approach to solving the 3D structures of lgt1/4Δ and lgt2Δ was to perform high temperature gas phase molecular dynamics simulations (HTMD), in order to search the accessible conformational space of the OS as completely as possible. Based on this conformational ensemble it was checked whether conformations could be extracted from the trajectory that most closely matched our NOE distance data. Selected structures were then used as starting structures for conventional MD simulations in explicit solvent using periodic boundary conditions (PBC) in order to study the dynamics and properties of the local conformational minima at room temperature. The aim in using MD simulations was to detect and characterize conformations that need to be present in the macroscopic molecular ensemble in order to explain the NOE data. This was performed by grouping the conformational data generated in explicit solvent into conformational sub-ensembles based on local minima taken from the conformational maps of the glycosidic linkages. For each sub-ensemble the effective H-H distances were calculated and compared to experimental NOE distances. This approach allowed us to solve the “conformational puzzle”, particularly by taking into account the “long-range” NOEs for lgt2Δ. In parallel we performed a distance mapping approach [30] in order to detect linkage conformations (φ/ψ torsions) in agreement with the measured “cross-linkage” NOEs restraints. In brief, the φ/ψ torsions of minimized disaccharides—representing the linkage types present in the OS—are rotated systematically and for each combination selected H-H distances are measured. The experimentally determined NOE distances are used as upper distance limits and are drawn as contours in a 2D graph. Intersecting NOE distance contours represent a likely conformation of the glycosidic linkage investigated. Additionally, conformational energy maps of the linkage—which can also be derived from the HTMD simulations—are displayed in the background in order to locate energy minima (for examples see Figure S1).

### 2.1. Conformational Analysis of lgt1/4Δ OS

Based on the conformational energy maps derived from the HTMD simulation (Figure S1) the α-d-Glc*p*-(1-5)-d-Kdo*p* (**A**-**Kdo**) linkage has a global minimum at φ/ψ (−10/40), with significant flexibility possible in the ψ dimension. Four local minima in the φ/ψ map are found for the linkages β-d-Glc*p*-(1-3)-α-d-Glc*p* (**B**-**A**) (φ/ψ/Δ*E*: 40/20/0, −30/−20/4.8, 35/175/4.5, 190/20/5.5) and β-d-Glc*p*-(1-4)-α-d-Glc*p* (**C**-**A**) (φ/ψ/Δ*E*: 45/10/0, 35/185/3.5, −20/−35/4.0, 170/0/7.0) and two φ/ψ minima for β-d-Glc*p*-(1-6)-α-d-Glc*p* (**E**-**A**) (φ/ψ/Δ*E*: 40/180/0, 185/185/4.0). All the side minima are predicted to have approximate relative energies >3 kcal/mol and should therefore not be populated significantly in the free state. Based on the energy maps lgt1/4Δ OS may therefore exist as a conformational ensemble where the linkage torsions are fluctuating around the φ/ψ global minimum. The distance mapping of the NOE restraints confirms this conclusion. Additionally, we compared directly the calculated average *r_eff_* from the HTMD ensemble with the experimental NOE constraints and found in general excellent agreement (despite the “non-physiological conditions” used for generating the ensemble) (Figure S2), which may be attributed to the relatively low populated side minima of the glycosidic linkages due to their high energy.

In order to access the flexibility of lgt1/4Δ at physiological conditions we performed several MD simulations at 310 K in explicit water solution (0.1% sodium chloride) using different starting structures. In total a timescale of two microseconds was sampled. The trajectories of the glycosidic linkage torsions are shown in Figure S3. Also in explicit solvent the linkage Glc*p*-(1-5)-d-Kdo*p* (**A**-**Kdo**) populates predominantly a single φ/ψ minimum (−20/40), but two closeby side minima (25/30 and −10/−40) also exist (Figure S4). Frequent transitions occur between the two syn states (A and B) for the β-d-Glc*p*-(1-3)-α-d-Glc*p* (**B**-**A**) and β-d-Glc*p*-(1-4)-α-d-Glc*p* (**C**-**A**) linkages, which means that the energy difference between minimum A and B is lower in the solvent simulations. It also becomes obvious from the MD simulation at 310 K that there are high energy barriers separating the anti states (C and D) from the syn states (A and B). A simulation started in minimum C of the β-d-Glc*p*-(1-4)-α-d-Glc*p* (**C**-**A**) linkage did not show any transition out of this state during a simulation time of 500 ns. A simulation started in minimum C of the β-d-Glc*p*-(1-3)-α-d-Glc*p* (**B**-**A**) linkage, however, showed a transition out of this state after 150 ns. It is obvious that it is technically very difficult to achieve a conformational equilibrium based on conventional MD simulations at 310 K in solvent—even on the microsecond time scale—particularly for a branched carbohydrate like lgt1/4Δ which can remain trapped in high-energy conformations for hundreds of nanoseconds.

**Figure 3:**
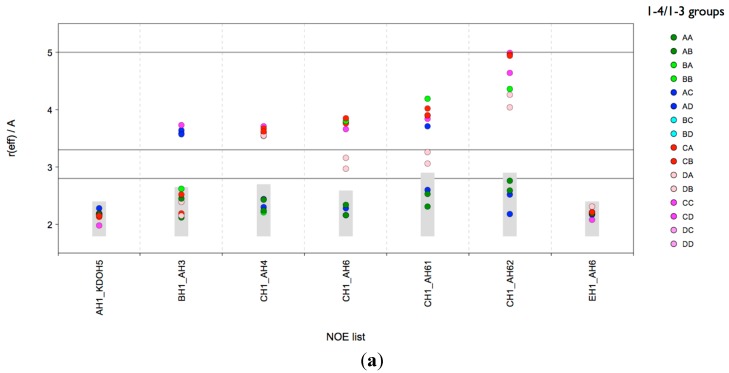

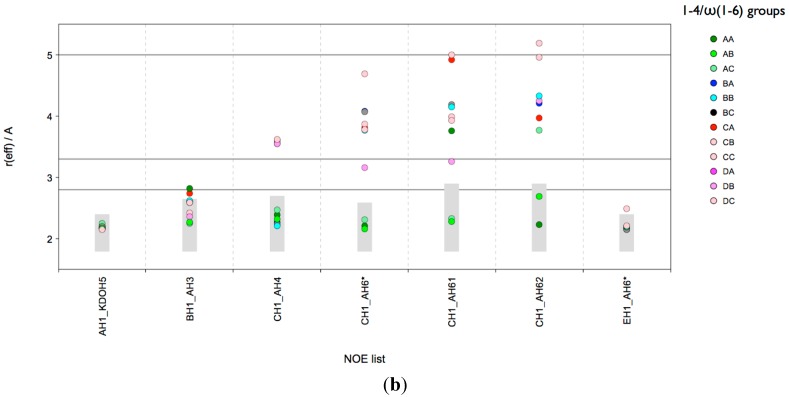
lgt1/4Δ OS: CAT analysis of *r_eff_* for conformational state groups. Combined states of the glycosidic linkages β-d-Glc*p*-(1-3)-α-d-Glc*p* (**B**-**A**) and β-d-Glc*p*-(1-4)-α-d-Glc*p* (**C**-**A**) (**a**); β-d-Glc*p*-(1-4)-α-d-Glc*p* (**C**-**A**) and ω torsion of β-d-Glc*p*-(1-6)-α-d-Glc*p* (**E**-**A**) (**b**). The horizontal lines indicate the distance limits for classification into strong (≤2.8 Å), medium (≤3.3 Å) and weak (≤5.0 Å) NOEs. The grey boxes indicate the range [*r*_vdW_, *r*_NOE_].

Consequently, the microsecond MD data does not represent a conformational equilibrium, and therefore no conclusions can be drawn on population distributions of the various conformers present. However, the data is suitable for an analysis of the NOE relevant H-H distances in the various local minima in order to predict their possible contributions to the measured NOE intensity. In order to perform such an analysis, the conformational snapshots of the MD ensemble were separated with the Conformational Analysis Tools (CAT) software into “conformational (state) groups” (sub-ensembles) and for each group the effective NOE distance (*r_eff_*) was calculated and compared to the NOE restraints found in the experiment. Such a grouping could be performed based on the local mininima found in the conformational maps. However for lgt1/4Δ there are 96 [1 × 4 × 4 × 2(φ/ψ) × 3(ω)] theoretical combinations of local minima (“conformational states”) possible, which would be already a rather challenging analysis. Therefore in order to reduce the complexity of the analysis we limited the separation to combined states of the β-d-Glc*p*-(1-3)-α-d-Glc*p* (**B**-**A**) and β-d-Glc*p*-(1-4)-α-d-Glc*p* (**C**-**A**) linkages only (compare also Figure S4). Each linkage has four minima (labels A, B, C and D) resulting in 16 combinations (AA, AB, *etc*.). For each combination a sub-ensemble was automatically generated on-the-fly when the trajectories were analysed with CAT. The results are shown in Figure 3a. It can be concluded that also in the solvent MD ensemble only those conformations could explain all NOEs that are in the A state of the β-d-Glc*p*-(1-4)-α-d-Glc*p* (**C**-**A**) linkage. The situtation is more ambiguous with respect to the β-d-Glc*p*-(1-3)-α-d-Glc*p* (**B**-**A**) linkage; in principle the A and B state would be in agreement with the NOEs. The medium NOE EH1-AH6x suggests that one of the H6 atoms has a distance of 2.9 Å from EH1. As shown in Figure 3b an AB state with a *gt* conformation of the ω torsion of the β-d-Glc*p*-(1-6)-α-d-Glc*p* (**E**-**A**) linkage would be in excellent agreement and the NOE EH1-AH6x would be assigned to AH62. This is also the outcome of the distance mapping analysis (Figure S1).

**Figure 4:**
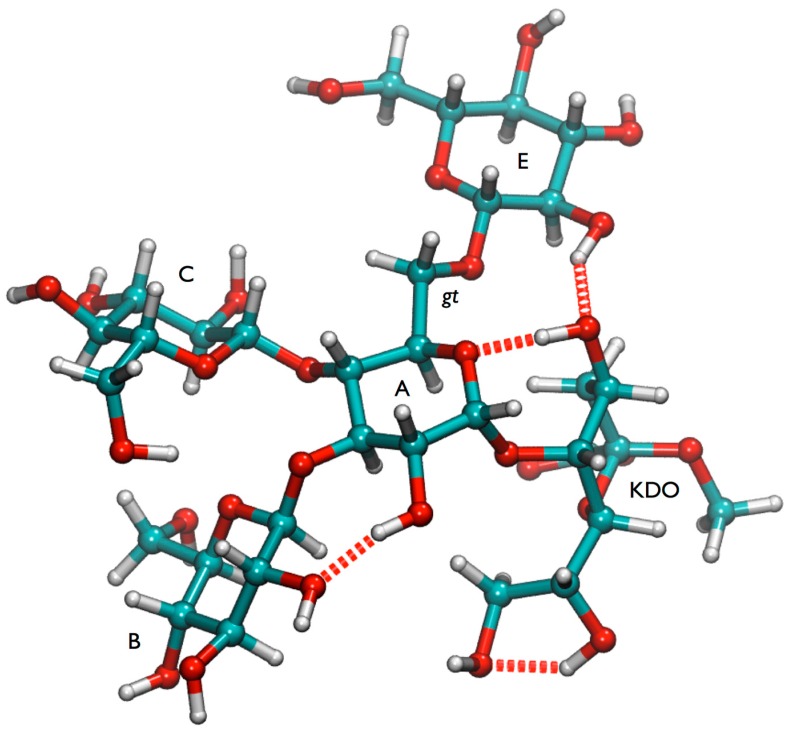
Global minimum conformation of lgt1/4Δ OS.

Based on the NOE data and the conformational analysis it can be concluded that the glycosidic torsions of lgt1/4Δ populate mainly the global minimum regions of the corresponding φ/ψ maps. Boltzmann population analysis of the HTMD ensemble revealed that the minima of the ω torsion of the (1-6) linkage −60° (*gg*), 60° (*gt*) and 160° (*tg*) have relative energies 0.7, 0.0, 2.7 kcal/mol, respectively. The NOE data also suggests that the *gt* minimum is more populated, which is in agreement with the energy analysis. The *gt* conformation is stabilized by several hydrogen bonds involving the Kdo residue (Figure 4 and Figure S5). However it cannot be ruled out that also the *gg* minimum is significantly populated at room temperature and the (1-6) linkage consequently may have some flexibility in lgt1/4Δ OS.

In order to further probe the flexibility of each of the glycosidic linkages of lgt1/4Δ OS a ^1^H T1 NMR analysis of the anomeric protons for each of the sugars in the OS was undertaken. The T1 data acquired in triplicate for each of the anomeric protons for lgt1/4Δ are shown in Table 4. This data highlights the flexibility observed with the 1-6 linkage in that it has a higher T1 value (T1 = 405 ms) than the other linkages, with the most restriction observed in the α-d-Glc*p*-(1-5)-d-Kdo*p* (**A**-**Kdo**) linkage (T1 = 295 ms) in accordance with the modeling observations.

**Table 4:** ^1^H-NMR T1 values (ms, 600 MHz, 298 K) for the anomeric protons of the *M. catarrhalis* OS mutants lgt1/4Δ, lgt4/5Δ and lgt5Δ.

Sugar Anomeric Proton and Linkage lgt1/4Δ	T1 Value (ms)
A: α-d-Glc*p*-(1-5)-d-Kdo*p* (**A-Kdo**)	295
B: β-d-Glc*p*-(1-3)-α-d-Glc*p* (**B-A**)	362
C: β-d-Glc*p*-(1-4)-α-d-Glc*p* (**C-A**)	335
E: β-d-Glc*p*-(1-6)-α-d-Glc*p* (**E-A**)	405
**Sugar Anomeric Proton and Linkage lgt4/5Δ**	
A: α-d-Glc*p*-(1-5)-d-Kdo*p* (**A-Kdo**)	273
B: β-d-Glc*p*-(1-3)-α-d-Glc*p* (**B-A**)	282
C: β-d-Glc*p*-(1-4)-α-d-Glc*p* (**C-A**)	302
E: β-d-Glc*p*-(1-6)-α-d-Glc*p* (**E-A**)	286
F: α-d-Glc*p*-(1-2)-β-d-Glc*p* (**F-E**)	285
α-d-Glc*p*-(1-2)-β-d-Glc*p* (t-Glc-**C**)	315
Terminal β-Gal*p*-(1-4)-α-d-Glc*p* (***t*Gal-F**)	330
**Sugar Anomeric Proton and Linkage lgt5Δ**	
A: α-d-Glc*p*-(1-5)-d-Kdo*p* (**A-Kdo**)	265
B: β-d-Glc*p*-(1-3)-α-d-Glc*p* (**B-A**)	275
C: β-d-Glc*p*-(1-4)-α-d-Glc*p* (**C-A**)	290
E: β-d-Glc*p*-(1-6)-α-d-Glc*p* (**E-A**)	294
F: α-d-GlcNAc*p*-(1-2)-β-d-Glc*p* (**F-E**)	294
D: α-d-GlcNAc*p*-(1-2)-β-d-Glc*p* (**D-C**)	323
Terminal β-Gal*p*-(1-4)-α-d-Glc*p* (***t*Gal-F**)	380

### 2.2. Conformational Analysis of lgt2Δ OS

The conformational maps for α-d-Glc*p*-(1-5)-d-Kdo*p*, β-d-Glc*p*-(1-3)-α-d-Glc*p* and β-d-Glc*p*-(1-6)-α-d-Glc*p* are very similar to the ones obtained for lgt1/4Δ, which means that the addition of the two monosaccharide rings do not impose significant spacial constraints on these glycosidic linkages (Figure S6). Only the energy map for the linkage β-d-Glc*p*-(1-4)-α-d-Glc*p* is significantly altered and a high-energy barrier is introduced between minimum A and B. However the location of the global minima is identical for lgt1/4Δ and lgt2Δ. The cross-linkage NOE restraints for lgt2Δ OS (Figure S7) are in agreement with conformations representing the global energy minimum for most of the linkages, but the possibility that some of the B minima are populated cannot be ruled out based on the cross-linkage NOE restraints. However there is clear evidence for a populated side minimum for the α-d-Glc*p*-(1-5)-d-Kdo*p* (**A**-**Kdo**) linkage*.* Additionally the situation for β-d-Glc*p*-(1-4)-α-d-Glc*p* (**C**-**A**) is ambiguous since the two NOE restraints could fit to minimum C, but also to a mixture of all three minima. In contrast to lgt1/4Δ only few NOE restraints are satisfied when comparing directly the calculated average *r_eff_* from the HTMD ensemble with the experimental NOE constraints of lgt2Δ. (Figure S8, top). Particularly the structures that would satisfy the long-range NOEs are clearly underrepresented in the ensemble. In general all the H-H distances for which long-range NOEs were found have a rather large distance fluctuation in the HTMD ensemble, consequently only a few should be in agreement with multiple narrow restraints imposed by the NOEs. Long-range NOEs depend on the local conformations of more than one glycosidic linkage (in contrast to cross-linkage NOEs), therefore the distance mapping method is not very suitable to determine conformations that are in agreement with long-range NOEs. Consequently the next step was to filter out structures that would satisfy distance restraints imposed by the long-range NOEs in order to get information on the glycosidic linkage conformations. No structures were obtained when all NOE restraints were used as a filter, therefore sub-ensembles were extracted based on subsets of restraints. The methyl group of β-d-Glc*p*NAc (**D**) is involved in five long-range NOEs and despite using a rather generous distance filter of 0.0–4.0 Å (0.0–4.5 Å for DCH3-EH1) only 74 frames out of 100,000 satisfied all the five distance filters applied simultaneously. The extracted structures all have an anti-ψ conformation (state C) of the β-d-Glc*p*-(1-4)-α-d-Glc*p* (**C**-**A**) linkage and are very similar in general (see overlayed 3D structures in Figure S8). For the ω torsion the *gg* state is more frequent than the gt state in the extracted DCH3-x sub-ensemble. From the DCH3-x sub-ensemble analysis it becomes evident that in this ensemble the BH1-CH2 distances are too long in order to satisfy the medium long-range NOE between the two H-atoms. Extracting frames with BH1-CH2 distances in the range 0.0–3.9 Å revealed that this NOE is likely caused by structures that have β-d-Glc*p*-(1-4)-α-d-Glc*p* (**C**-**A**) in state A and β-d-Glc*p*-(1-3)-α-d-Glc*p* (**B**-**A**) in state B (data not shown). Additionally, the extracted DCH3-x sub-ensemble doesn’t satisfy the long-range NOE FH2-DH2 very well. Extracting frames with a FH2-DH2 distance range 0.0–3.9 Å revealed that the structures also have an anti-ψ conformation (state C) of the β-d-Glc*p*-(1-4)-α-d-Glc*p* (**C**-**A**) linkage, but the long-range NOE DCH3-EH1 is no longer satisfied. In conclusion the preliminary analysis based on the HTMD ensemble reveals that multiple conformations of lgt2Δ need to be present in order to explain all the long-range NOEs.

**Figure 5:**
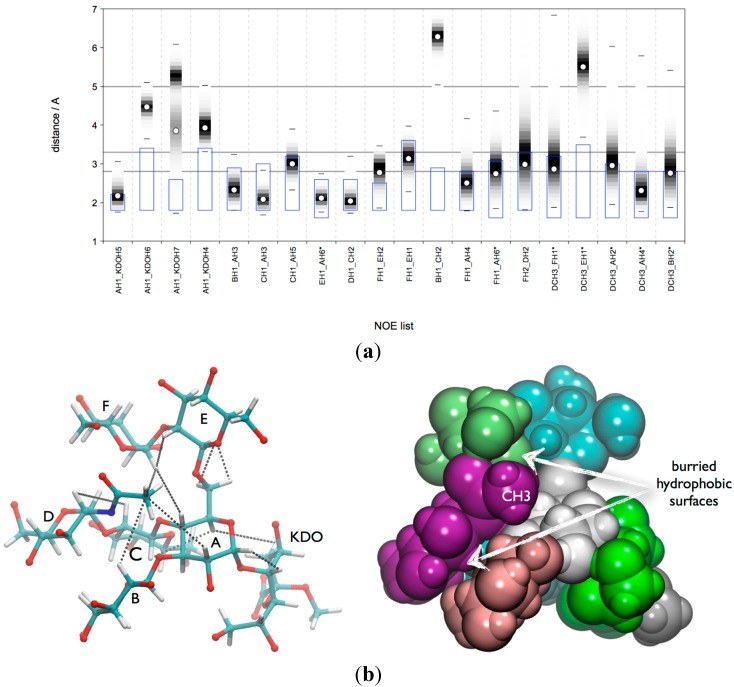
lgt2Δ OS: (**a**) Comparison of the calculated *r_eff_* derived from a 300 ns MD simulation at 300 K of the (1-4)anti-ψ(1-6)*gg* conformation with the experimental NOE restraints. Calculated average *r_eff_* is shown as white circle; histogram of the distances is shown in the background with darker shading meaning higher population. The experimental NOE restraints are indicated with a blue box; (**b**) A representative 3D structure of the (1-4)anti-ψ(1-6)*gg* conformation. The fulfilled NOE restraints are indicated as dotted lines (**Left**). Space filling model (**Right**).

One of the most interesting results derived from the HTMD analysis is the possible existence of a “(1-4)anti-ψ(1-6)*gg*” conformation of lgt2Δ OS. In order to confirm the stability and characterize such a conformation in more detail an MD simulation in explicit solvent was performed. We sampled 300 ns at 300 K and the structure was completely stable during the whole simulation, even the ω torsion remained in the *gg* state (data not shown). The reason for this stability might be the existence of several inter-residue hydrogen bonds (Figure S9), but also very intensive hydrophobic contacts between the Glc*N*Ac (**D**) and the terminal glucoses of the 1-3 (**B**) and 1-6 (**F**) branches (Figure 5). The latter interaction should contribute favorably to the free energy of the conformation in solution, since it effectively reduces the amount of water accessible hydrophobic surfaces in the system with the consequence that water molecules would be released into the bulk solvent, which should increase the entropy of the system.

However, a comparison of the ensemble *r_eff_* with the experimental long-range NOEs confirmed that this conformation alone cannot account for all of the NOEs observed (Figure 5a). The distances for BH1-CH2 and DCH3-EH1 are too large for explaining the measured NOE intensity. Also the *r_eff_* for some cross-linkage NOEs are probably too short for the measured NOE intensities. Consequently there are very likely other conformations present next to “(1-4)anti-ψ(1-6)*gg*”.

**Figure 6:**
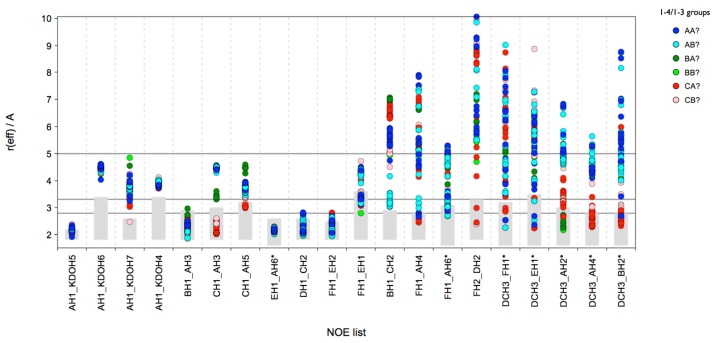
lgt2Δ OS: CAT analysis of *r_eff_* for conformational sub-ensembles (state groups). Combined states of all glycosidic linkages except the (1-5)-linkage to Kdo are used. The horizontal lines indicate the distance limits for classification into strong (≤2.8 Å), medium (≤3.3 Å) and weak (≤5.0 Å) NOEs. The grey boxes indicate the range [*r*_vdW_, *r*_NOE_]. The color code separates sub-ensemble types defined by the (1-4)- and (1-3)-linkages: “AB?” means the (1-4)-linkage is in minimum A and the (1-3)-linkage is in minimum B; the states of the other linkages are not considered for color coding indicated by the “?”. “AB?” could therefore be state groups ABAAA, ABABB, ABBAA, *etc.* The composite “virtual distances” are marked with an asterisk (*).

In order to generate a conformational ensemble that could be used for a comprehensive analysis we sampled (accumulated) 3.7 μs at 310 K in explicit solvent. Because the lifetime of many conformational states was >100 ns—as observed in the 300 K MD—different starting structures were used. The trajectories of the glycosidic torsions are shown in Figure S10. As described for lgt1/4Δ OS such an accumulated MD trajectory can be used for a CAT analysis of *r_eff_* for individual conformational state groups (sub-ensembles). In order to facilitate the interpretation of the results, the number of state variables (e.g., linkages) that are combined should be kept small, so we limited the linkage states (A, B, C) used to those that fit to experimental restraints in the distance mapping (Figure S7) and excluded the (1-5)-linkage. The state groups were defined by combining the following conformational minima (compare Figure S11): A, B and C of the (1-4) linkage (**C-A**), A and B of the (1-3) linkage (**B-A**), A(*gg*), B(*gt*) and C(*tg*) of the (1-6) linkage (**E-A**) (only the main φ/ψ-minimum A at 40/170 was used), A, B and D of the (1-2) linkage (**D-C**), A and B of the (1-2) linkage (**F-E**). The conformational state groups (five linkages, 108 theoretical combinations: AAAAA, AAAAB, AAABA, *etc*.) were used to group the MD snapshots into conformational sub-ensembles. For each sub-ensemble, *r_eff_* was calculated for all distances for which H-NOEs were experimentally observed. In total 69 sub-ensembles were populated in the MD data and the results are shown in Figure 6. It is evident that FH2-DH2 is a reporter NOE for “(1-4)anti-ψ” conformations (represented by a state group pattern “C????”, ? is used as wild card meaning “any” linkage state) because *r_eff_* within the limits of the NOE restraint are only found if the (1-4)-linkage is in state C (anti-ψ). However, the connecting graph between FH2 and DH2 contains nine rotatable bonds, consequently additional conditions need to be met in order to bring the two protons into a distance of about 2.9 Å. It should be noted that also many other NOEs would be in agreement with anti-ψ states of the (1-4)-linkage. The previous analysis of the 300 K MD ensemble revealed that the long-range NOEs BH1-CH2 and DCH3-EH1cannot be explained by such a “(1-4)anti-ψ(1-6)*gg*” conformation, and the question arises whether the sub-ensemble analysis can shed some light into which other conformations need to be present in order to explain the NOE data. Based on the sub-ensemble analysis the medium NOE BH1-CH2 is very likely caused by sub-ensembles of state group pattern “AB???”, which have the (1-4)-linkage in state A and the (1-3)-linkage in state B. However, for (1-4)syn states, one would expect an NOE CH1-AH4 present in the spectrum, which could not be detected unfortunately. Since also the NOE DCH3-EH1 could originate from (1-4)syn states there is some probability that (1-4)syn states are present despite the missing cross-linkage NOE CH1-AH4. When the (1-4)-linkage is in the A state, the (1-3)-linkage becomes more flexible and frequent transitions between A and B states occur (Figure S10). This would also give some additional support that “AB???” type conformations may exist.

**Figure 7:**
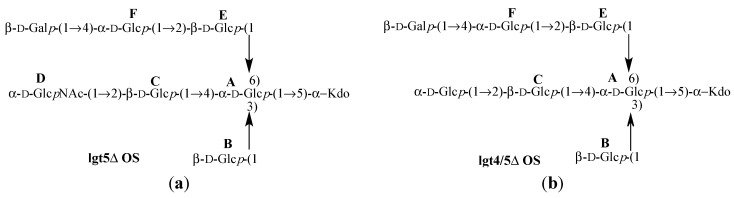
Structures of *Moraxella catarrhalis* serotype A (strain 2951) (**a**) lgt5Δ OS [14] and (**b**) lgt4/5Δ OS [15].

Again, the flexibility of the linkages were probed using T1 analysis and two other OS mutants (lgt4/5Δ and lgt5Δ, Table 4, Figure 7) were compared to lgt1/4Δ where each had an additional sugar on the 1-6 linkage (β-d-Gal*p*-(1-4)-**F**). In the case of lgt4/5Δ OS it has a terminal α-d-Glc*p* in place of residue **D** to investigate whether the Glc*N*Ac in Lgt2Δ OS had any bearing on the flexibilty and conformations observed. This mutant also has biological relevance since this residue is a Glc*N*Ac in serotype A *M. catarrhalis*, but a Glc in serotype B. There is good agreement between the T1 of the anomeric protons of lgt4/5Δ and lgt5Δ OS, and overall much less flexibility is observed in equivalent linkages in lgt1/4Δ OS.

## 3. Experimental Section

### 3.1. Bacterial Strains and Isolation of OS

*M. catarrhalis* strains 2951lgt2Δ, 2951lgt1/4Δ, 2951lgt5Δ and 2951lgt4/5Δ were grown in glucose supplemented BHI media. LOS was isolated using a modified [15] phenol/chloroform/petroleum ether extraction method. The OS was obtained after mild acid hydrolysis to yield OS described herein as lgt2Δ, lgt1/4Δ, lgt5Δ and lgt4/5Δ as previously described [15].

### 3.2. NMR

Oligosaccharide (1 mg) dissolved in D_2_O (CIL 99.998%) was lyophilized in triplicate to remove exchangeable protons. The samples were dissolved in 750 µL of D_2_O under nitrogen and ^1^H and ^13^C spectra were recorded at 298 K on a Bruker Avance spectrometer operating at 600 MHz and 150 MHz, respectively. Chemical shifts are reported in ppm referenced to internal acetone (^1^H 2.225 ppm, ^13^C 31.45 ppm). 2D ^1^H-NOESY and 2D ^1^H^13^C-HSQC-NOESY spectra of OS were acquired with 256 scans and a minimum of 512 slices at 298 K and 600 MHz with a NOESY mixing times of 200 and 400 ms. NOEs (from the 200 ms spectrum) were integrated using standard Bruker software and categorized using the distance constraints: strong 1.8–2.8 Å, medium 1.8–3.3 Å and weak 1.8–5.0 Å. NOE peaks that involve CH_3_ or CH_2_ groups are classified as “composite” NOEs. The NOE distance *r*_NOE_ calculated from the peak integral *I* using equation *r*_NOE_ *= r_ref_* (*I_ref_* /*I*)^1/6^ (reference distance *r_ref_* = 2.46 Å assigned to the distance between H1 and H2 protons of glucose A where *I_ref_* = 514 for lgt2Δ and *I_ref_* = 344 for lgt1/4Δ). The reference distance 2.46 Å was measured in Cambridge Structural Database entry MALTOS11.

It should be noted that *r*_NOE_ distances calculated for composite NOEs are “virtual distances” that are shorter than any of the “real distances” between the contributing H-H pairs.

^1^H T1 values were acquired with the Inversion Recovery method using standard pulse sequences from the Bruker library. T1 values were acquired in triplicate with 1 K scans and a list of 10 variable delays ranging from 0.1 s to 2 s. An acquisition delay of 5 times the longest T1 value measured was used in the acquisition of the final spectra for analysis. All spectra were acquired, processed and analysed using standard Topspin software on a Bruker Avance spectrometer operating at a ^1^H frequency of 600.13 MHz at 298 K.

### 3.3. Molecular Modeling

High-temperature molecular dynamics (HTMD) simulations were performed at 700 K in the gas phase for 100 ns using the MM3 force field [31] as implemented in the TINKER software [32]. The carboxyl-group of the KDO was protonated in order to avoid potential artifacts caused by a charged molecule. A dielectric constant of 4 was used and torsion restraints were applied on the ring torsions to avoid inversion of the carbohydrate rings during MD. The integration time step was 1.0 fs. Snapshots were recorded every 1 ps resulting in a conformational ensemble consisting of 100,000 frames. Atom-type assignment and analysis of the data (including generation of most of the scientific plots) was performed using Conformational Analysis Tools (CAT) [33]. Conformational maps were calculated (as described in [34]) in order to check whether the accessible conformational space of the glycosidic linkages was sufficiently explored and also to be displayed in the background for the distance mapping method [30]. CAT can calculate “virtual NOE distances” on-the-fly for CH_2_ and CH_3_ groups from 3D structures, therefore the experimental NOE distances calculated for the composite NOEs could be used directly for the comparison with modeled conformations and a “pseudo-atom correction” (+1 Å) was not necessary. The glycosidic torsions are defined as: φ = H1-C1-Ox-Cx-Hx, ψ = H1-C1-Ox-Cx-Hx (x = linkage position), ω = O5-C5-C6-O6. VMD [35] was used for displaying and animating trajectories.

The MD simulations in explicit solvent were set up and performed using YASARA [36]. The carbohydrate was solvated in a box of TIP3 water, which was 10 Å larger on each side than the dimensions of the solute. The concentration of added sodium chloride ions was 0.1%. Pressure control was performed by scaling the periodic simulation box in order to keep the water density at 0.996 g/L. The default time step was used which is 2.5 fs. The GLYCAM [37] force field parameters were used for the carbohydrates. The missing parameters for Kdo were derived from GAFF [38] and AM1-BCC charge calculations [39] using the AutoSMILES method implemented in YASARA. Long-range Coulomb interactions were calculated using the Particle Mesh Ewald (PME) algorithm. Simulations were performed at 310 K using different starting structures for both OS for an accumulated simulation time of 2 µs (lgt1/4Δ) and 3.7 µs (lgt1/4Δ) with snapshots recorded every 25 ps. Using 4 CPUs per simulation the sampling rate was about 45 ns/day. An additional MD simulation of the conformation “(1-4)anti-ψ(1-6)*gg*” was performed at 300 K for 300 ns.

For comparison of the simulation data with the experimental NOE distance restraints the average effective H-H distance *r_eff_* was calculated from distances *r* measured for the frames in the (sub-) ensembles using the Equation (1):

(1) $$r_{eff}={\left(\frac{N}{\sum_{1}^{N}r^{-6}}\right)}^{1/6}$$

*N* = number of frames, *r* = H-H distance.

Composite “virtual” distances r^*^ for CH_2_ and CH_3_ groups were calculated using Equation (2):

(2) $$r^{*}={\left(\overset{N}{\underset{i=1}{\sum }}{r_{i}}^{-6}\right)}^{-1/6}$$

*N* = number of H-H pairs, *r_i_* = distance of H-H pair *i*.

## 4. Conclusions

**Figure 8:**
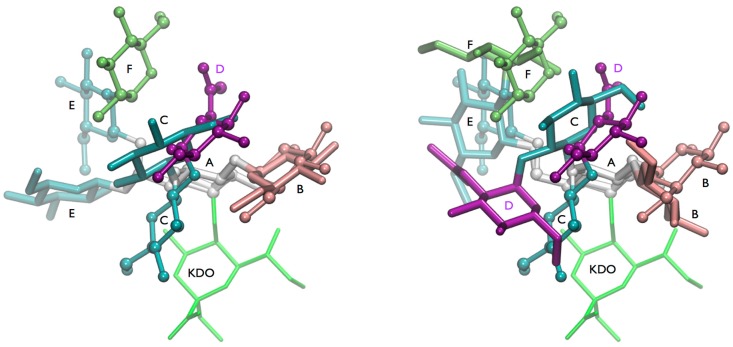
Overview on conformations found for the highly branched glucose-rich inner core of lipooligosaccharide of *M. catarrhalis*. (**Left**) Comparison of the energy minimum conformation of lgt1/4Δ OS (stick) and the “(1-4)anti-ψ(1-6)*gg*” conformation of lgt2Δ OS (cpk); (**Right**) Comparison of the “(1-4)anti-ψ(1-6)*gg*” conformation (cpk) of lgt2Δ OS and a conformation that has the (1-4)-linkage in minimum A and the (1-3)-linkage in minimum B. The terminal GlcNAc residue (D) is shown in purple.

Compared to the lgt1/4Δ OS structure, the addition of two sugar residues to the (1-4) and (1-6) chains caused a major conformational change in the three-dimensional structure of the lgt2Δ OS (Figure 8). The (1-3), (1-4), and (1-6) glycosidic linkages from the central α-d-Glc*p* (**A**) have significantly altered preferences between the two structures based on the NOE data. In lgt1/4Δ OS the (1-4) linkage mainly populates the syn minimum (A) on the conformational map, whereas in lgt2Δ OS anti-ψ (C) is probably the most populated state. The (1-3) linkage seems to be mainly in the global minimum in both OS, but there is some evidence that the B state might also be populated. The (1-6) linkages are generally known to be flexible, and also for lgt1/4Δ OS it can be assumed that there is a conformational equilibrium mainly between the *gg* and *gt* states of the ω torsion. The T1 data are in agreement with these data and show a high degree of flexibility for the lgt1/4Δ OS. However, for lgt2Δ OS, a significant increase of the lifetime for the (1-4)anti-ψ(1-6)*gg* conformation was found due to intensive interactions of the (1-6) branch with the Glc*pN*Ac residue (**D**). Overall, this lgt2Δ OS conformation is very compact, with the **D** residue folding back towards the center of the molecule. The **F**, **D**, and **B** sugar rings are almost stacked onto one another, allowing very little freedom for the (1-4) branch. This is also shown in the reduced accessible conformational space in the free energy map for the (1-4) linkage of lgt2Δ OS compared to lgt1/4Δ. It is interesting to note that the 4′ positions of residues **F** and **D** are pointing out and away from the molecule, leaving them available for further extension without disrupting the current 3D structure. This explains why larger structures extended from these positions do not exhibit further ^1^H-NMR chemical shift changes, which would indicate further conformational changes [20]. Despite some shortcomings in the matching of some NOE intensities, it can be concluded that the “(1-4)anti-ψ(1-6)*gg*” conformation is likely to be the dominant conformation of lgt2Δ OS, which is remarkable due the fact that anti-ψ states are usually high-energy states and have rarely been detected experimentally. The unusual conformational preference of lgt2Δ OS (and larger structures) could be explained by an intensive hydrophobic interaction between residues **D** and **F**, both of which are not present in lgt1/4Δ.

The (1-5) glycosidic linkage between residue **A** and Kdo is the only linkage that is consistent between the two structures (lgt1/4Δ and lgt2Δ OS). This is interesting as each linkage that underwent a conformational change caused a corresponding variation in the ^1^H-NMR chemical shift of the associated anomeric proton, while the unchanged linkage (**A**-**Kdo**) had no impact on the anomeric proton chemical shift. This highlights the value of using anomeric proton chemical shifts as indicators for conformational changes in glycosidic linkages of OS.

The occurrence of a significant conformational change between a truncated and extended OS structure may have important implications on the strategy utilized to design carbohydrate vaccines composed of truncated OS that, although they may contain the core residues common to many strains, may not necessary possess the same three-dimensional structure as a wild type OS. Additionally, the potential for OSs to twist into a defined secondary structure is an important consideration for structure-based drug design, such as targeting an OS to a specific protein binding site. Accordingly, a significant and otherwise unexplained change in anomeric proton chemical shifts could be utilized as an early indicator that a conformational change is occurring during an OS synthesis.

## Supplementary Materials

Supplementary materials can be accessed at: http://www.mdpi.com/1420-3049/20/08/14234/s1.
