# Supplementary material for: An Unusual Carbohydrate Conformation is Evident in Moraxella catarrhalis Oligosaccharides
Source: Molecules. 2015 Aug 5;20(8):14234–53. doi: 10.3390/molecules200814234 (PMC6332130; doi:10.3390/molecules200814234)
Supplement: Supplementary file 1 [file molecules-20-14234-s001.pdf]

Supplementary

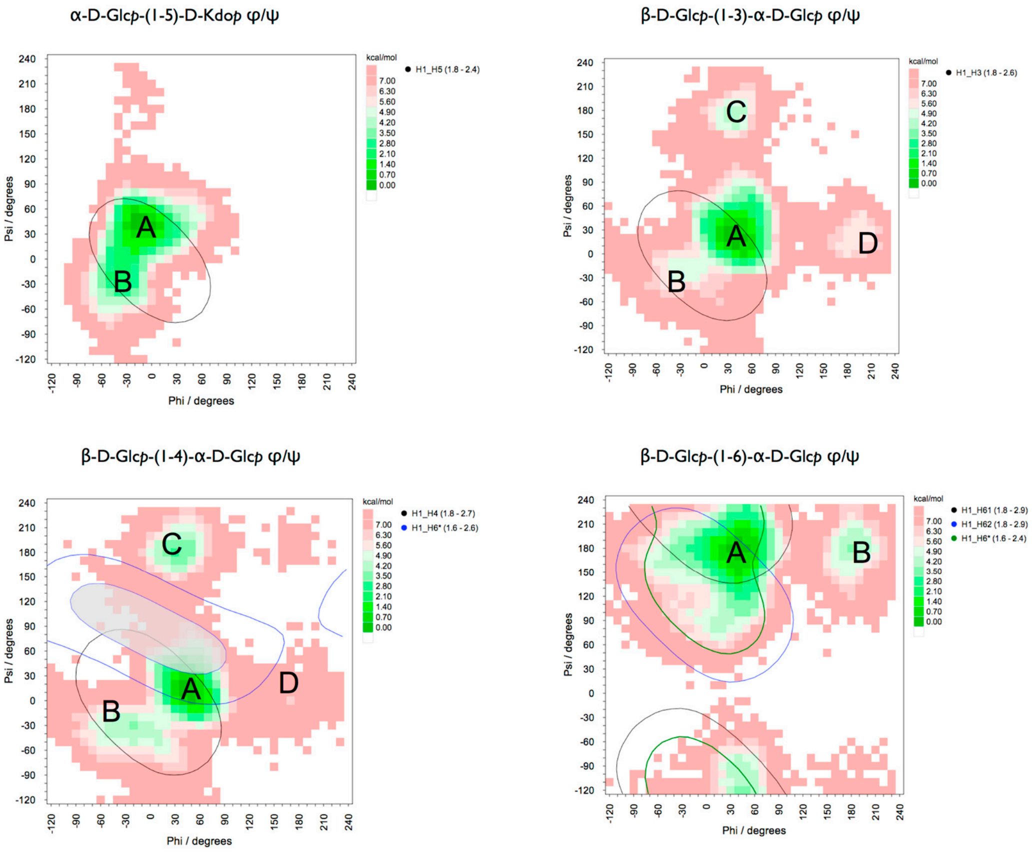

Figure S1. Distance mapping of glycosidic linkages of lgt1/4Δ OS using NOE distances as upper restraints. A van-der-Waals distance of 1.8 Å is used as a lower distance restraint which results in some areas that are not accessible (shown in light grey). For composite NOEs—marked with an asterisk (*)—the calculated virtual distance is used as upper restraint and 1.6 Å—the virtual distance when both distances (H61-H1 and H62-H1) are 1.8 Å—is used as lower distance restraint. Conformational free energy maps derived from 100 ns HTMD (700 K) are displayed in the background with the local minima labeled.

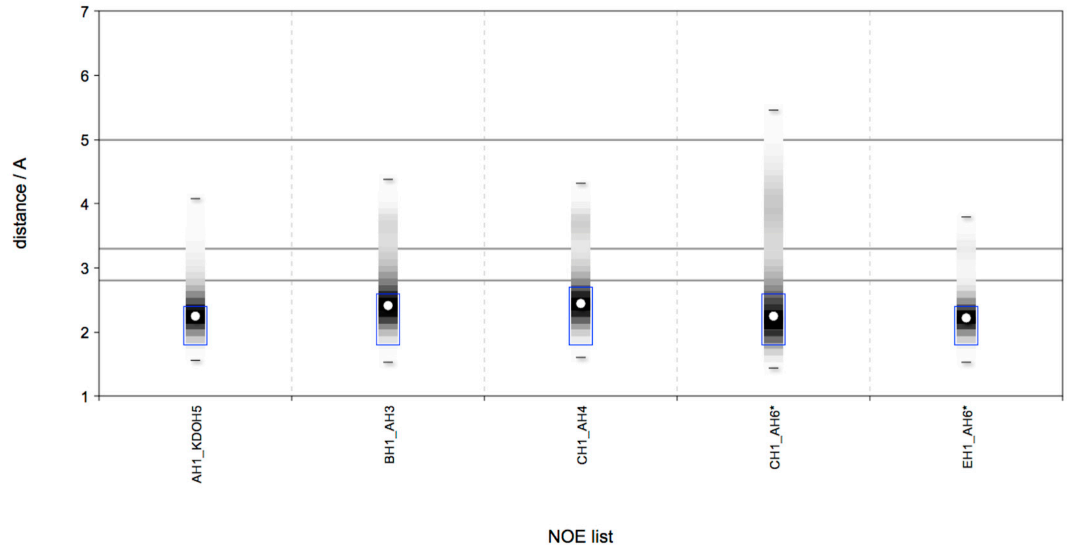

Figure S2. lgt1/4Δ OS: Comparison of the H-H distance statistics of the HTMD ensemble with the experimental NOE restraints in order to check whether the generated ensemble contained conformations that would satisfy the short distance restraints imposed by the strong NOEs. Calculated ensemble average r_{eff} is shown as white circle, histogram of the distances is shown in the background with darker shading meaning higher population. The experimental NOE restraints are indicated with a blue box. The composite "virtual distances" are marked with an asterisk (*). It should be noted that the ensemble was derived at 700 K in the gas phase and the NMR measurements were performed at 298 K in solvent so there is no physical justification to compare the ensemble statistics directly. That a reasonable agreement was found for all r_{eff} with the corresponding experimental NOE distance restraints cannot be generalized, but is however remarkable.

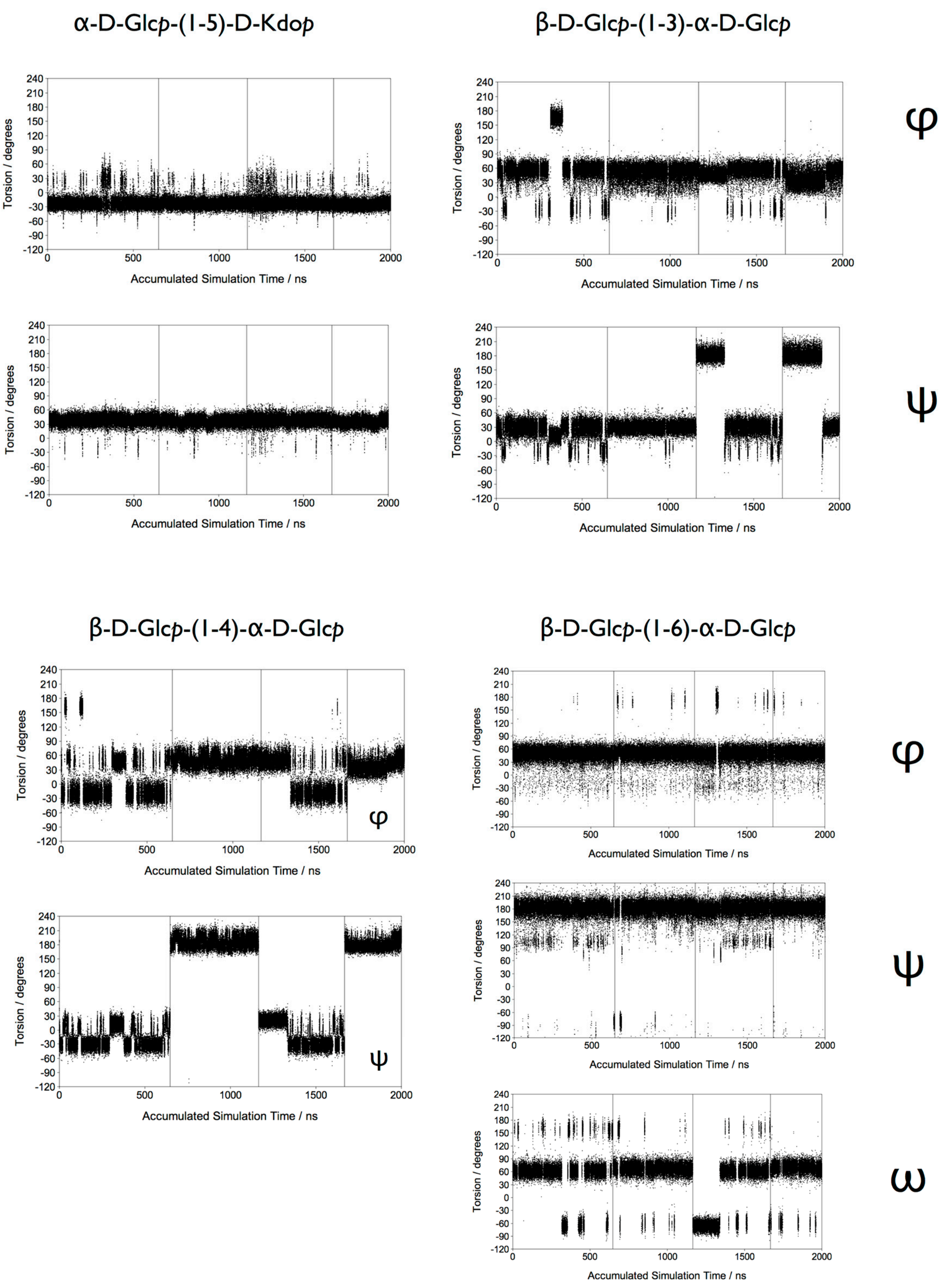

Figure S3. lgt1/4Δ OS: Dynamics of the glycosidic torsions on the microsecond time scale in explicit solvent at 310 K. Four independent trajectories were sampled using different starting structures. For efficient display the trajectories are shown as single continuous plots. The different MD runs are separated by a vertical line.

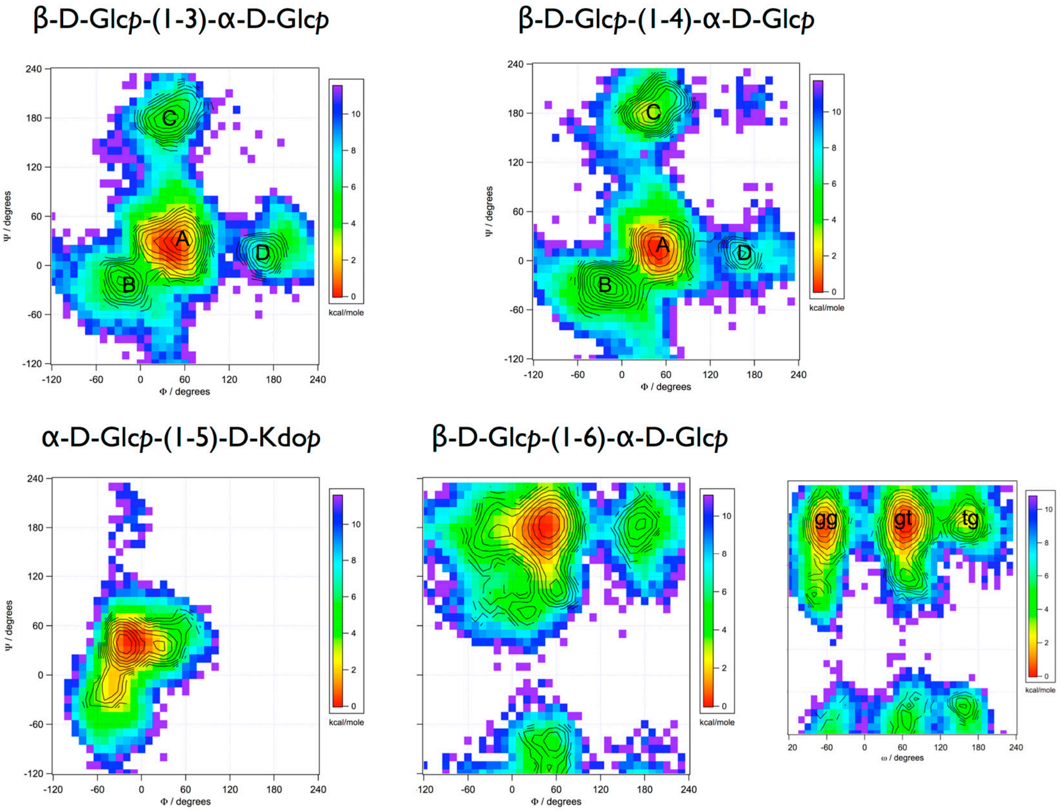

Figure S4. lgt1/4Δ OS: Local minima found for the glycosidic linkages from MD simulations in explicit solvent at 310 K (shown as contours). The data is calculated from four independent trajectories. It should be noted that the relative population between the local minima is artificial since no conformational equilibrium between the states has been sampled. The location of the minima in explicit solvent is very similar to their respective location in the free energy map (colored background) calculated based on HTMD simulation.

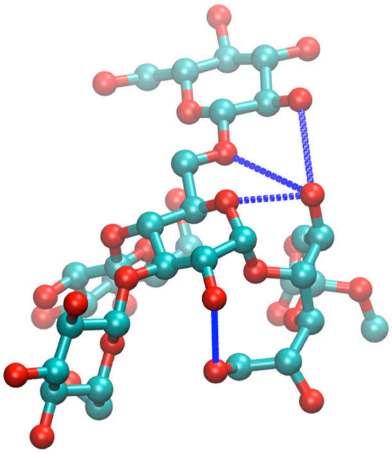

Hydrogen bond analysis

Total number of H-bonds populated = 82

Index	Donor	Acceptor	Population	Distance	Angle
1	KDO:O4	A:O5	88.3	2.75	158.35
2	E:O2	KDO:O4	28.7	2.86	161.49
3	KDO:O8H	A:O2	25.4	2.84	160.74
4	KDO:O4	A:O6	14.0	3.01	130.47
5	A:O2	KDO:O8H	11.0	2.83	157.87
6	E:O6	C:O2	9.2	2.90	150.47
7	KDO:O8H	B:O6	9.2	2.83	160.36
8	A:O2	KDO:O7H	9.1	2.80	159.73
9	C:O6	B:O5	8.6	2.95	141.94
10	C:O2	E:O6	8.5	2.80	160.58
11	A:O2	B:O2	8.4	2.80	165.13
12	C:O6	C:O5	7.8	2.62	124.48
13	B:O6	KDO:O8H	6.5	2.84	158.52
14	B:O2	C:O6	5.5	2.84	153.78
15	KDO:O7H	A:O2	5.3	2.88	153.24
16	B:O6	B:O5	5.2	2.61	123.96

Figure S5. lgt1/4Δ OS: Intra-molecular hydrogen bond statistics for extracted frames in the global minimum of linkages β-D-Glc*p*-(1-3)-α-D-Glc*p* and β-D-Glc*p*-(1-4)-α-D-Glc*p*. Only H-bonds with population above 5% are shown in the table and only those with population above 10% are indicated with a blue dotted line in the 3D graph. The average number of H-bonds is 3.0 (a geometric H-bond criterium was used: D-A distance ≤ 3.2 Å, D-H-A angle ≥ 120°). It should be noted that the inter-residue hydrogen bond between Kdo:O4 and A:O5 is not possible in the complete *M. catarrhalis* LOS because Kdo:O4 is 2-4 linked to another Kdo residue.

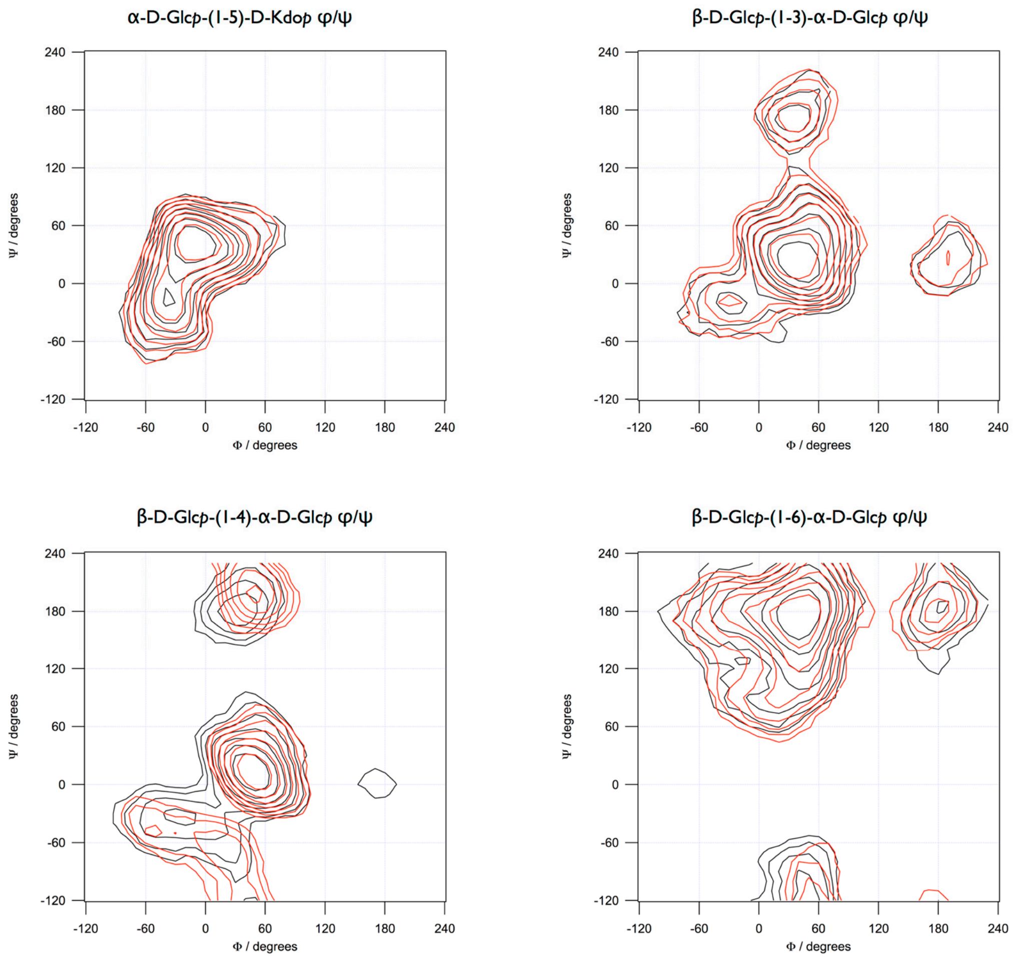

Figure S6. Overlay of the conformational free energy maps derived from 100 ns HTMD (700 K) for lgt1/4Δ OS (black contours) and lgt2Δ OS (red contours). Contours are shown in steps of 1 kcal/mol up to a maximum of 7 kcal/mol above the global minimum.

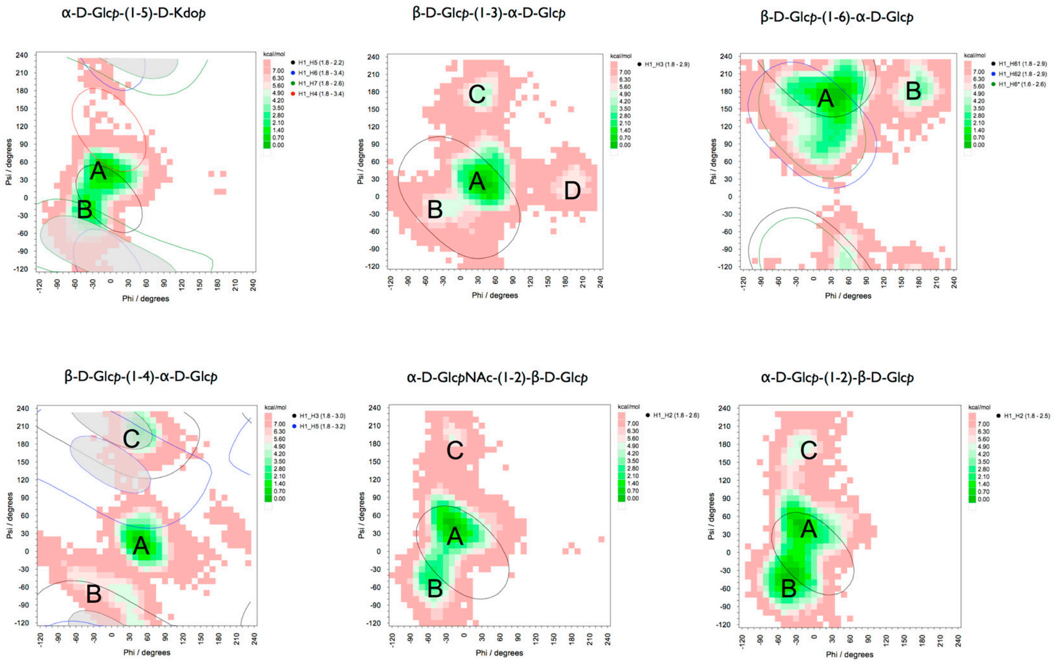

Figure S7. Distance mapping of glycosidic linkages of lgt2Δ OS using NOE distances as upper restraints. Conformational free energy maps derived from 100 ns HTMD (700 K) are displayed in the background with the local minima labeled.

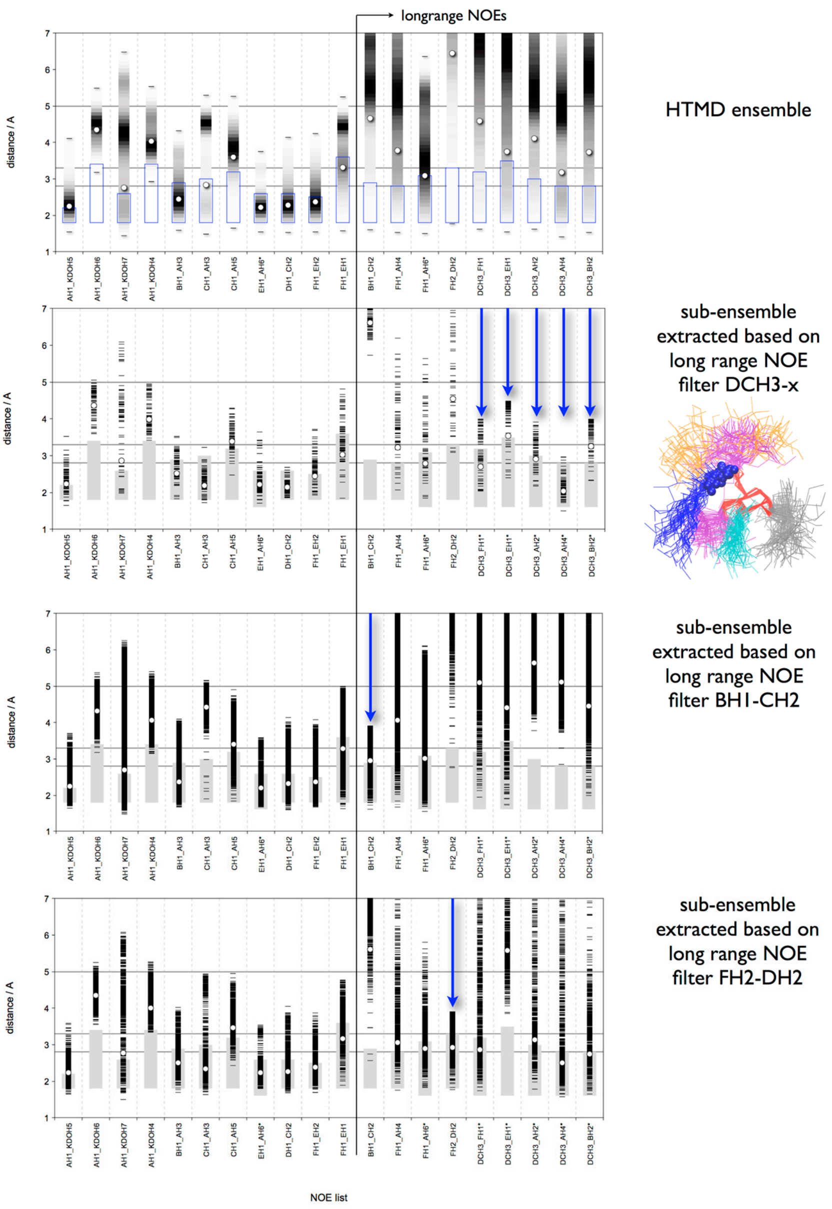

Figure S8. lgt2Δ OS: Comparison of the distance statistics of the HTMD ensemble with the experimental NOE restraints. See also text in Figure S2. Calculated average r_{eff} is shown as white circle, histogram of the distances is shown in the background with darker shading meaning higher population. The experimental NOE restraints are indicated with blue/grey boxes. The results for the original HTMD conformational ensemble are shown on the top. A sub-ensemble consisting of 74 frames was extracted using five distance filters representing long-range NOEs of the methyl group of the β-D-Glc*p*NAc (indicated by the blue arrows). The individual distances found in the sub-ensemble are indicated in the graph on the bottom as small horizontal lines. The sub-ensemble is also shown in 3D. The structures were superimposed on the central glucose residue. Analysis of sub-ensembles satisfying NOEs BH1-CH2 and FH2-DH2 are also shown.

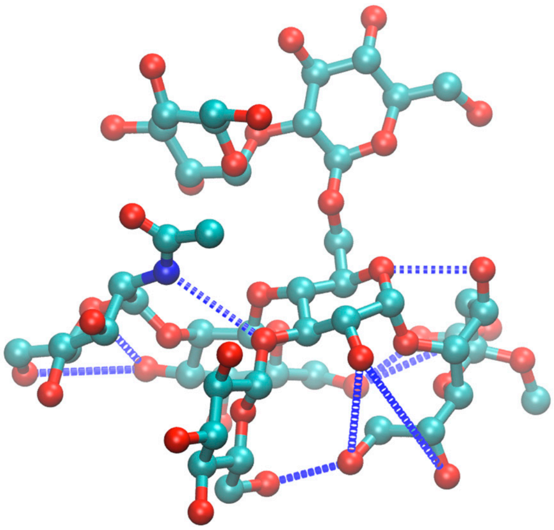

Hydrogen bond analysis

Total number of H-bonds populated = 58

Index	Donor	Acceptor	Population	Distance	Angle
1	KDO:O4	A:O5	71.4	2.78	162.33
2	D:N2	A:O3	45.9	3.05	154.17
3	A:O2	KDO:O7H	41.2	2.77	163.49
4	C:O3	D:O5	41.1	2.89	150.37
5	C:O6	KDO:O1H	32.5	2.73	162.34
6	C:O6	KDO:O1B	27.0	2.73	161.71
7	C:O3	D:O6	22.7	2.97	137.18
8	D:O6	C:O3	22.4	2.87	155.18
9	B:O6	KDO:O8H	17.6	2.81	161.07
10	KDO:O8H	A:O2	12.4	2.87	156.20
11	KDO:O7H	A:O2	8.9	2.85	152.83
12	A:O2	B:O2	8.3	2.81	163.52
13	KDO:O8H	B:O6	8.0	2.79	162.88
14	D:N2	A:O4	5.9	3.13	136.58
15	B:O2	A:O2	5.1	2.87	159.31
16	B:O6	C:O2	4.7	2.98	153.16
17	A:O2	KDO:O8H	4.2	2.82	155.90
18	F:O6	D:O2N	3.8	2.83	151.69
19	F:O2	A:O4	1.5	2.84	158.46

Figure S9. lgt2Δ OS: Hydrogen bond analysis of the "(1-4)anti-ψ(1-6)*gg*" conformation based on 300 ns MD simulation in explicit solvent at 300 K. Only H-bonds with population above 5% are shown in the table and only those with population above 10% are indicated with a blue dotted line in the 3D graph. It should be noted that the inter-residue hydrogen bond between Kdo:O4 and A:O5 is not possible in the complete *M. catarrhalis* LOS because Kdo:O4 is 2-4 linked to another Kdo residue.

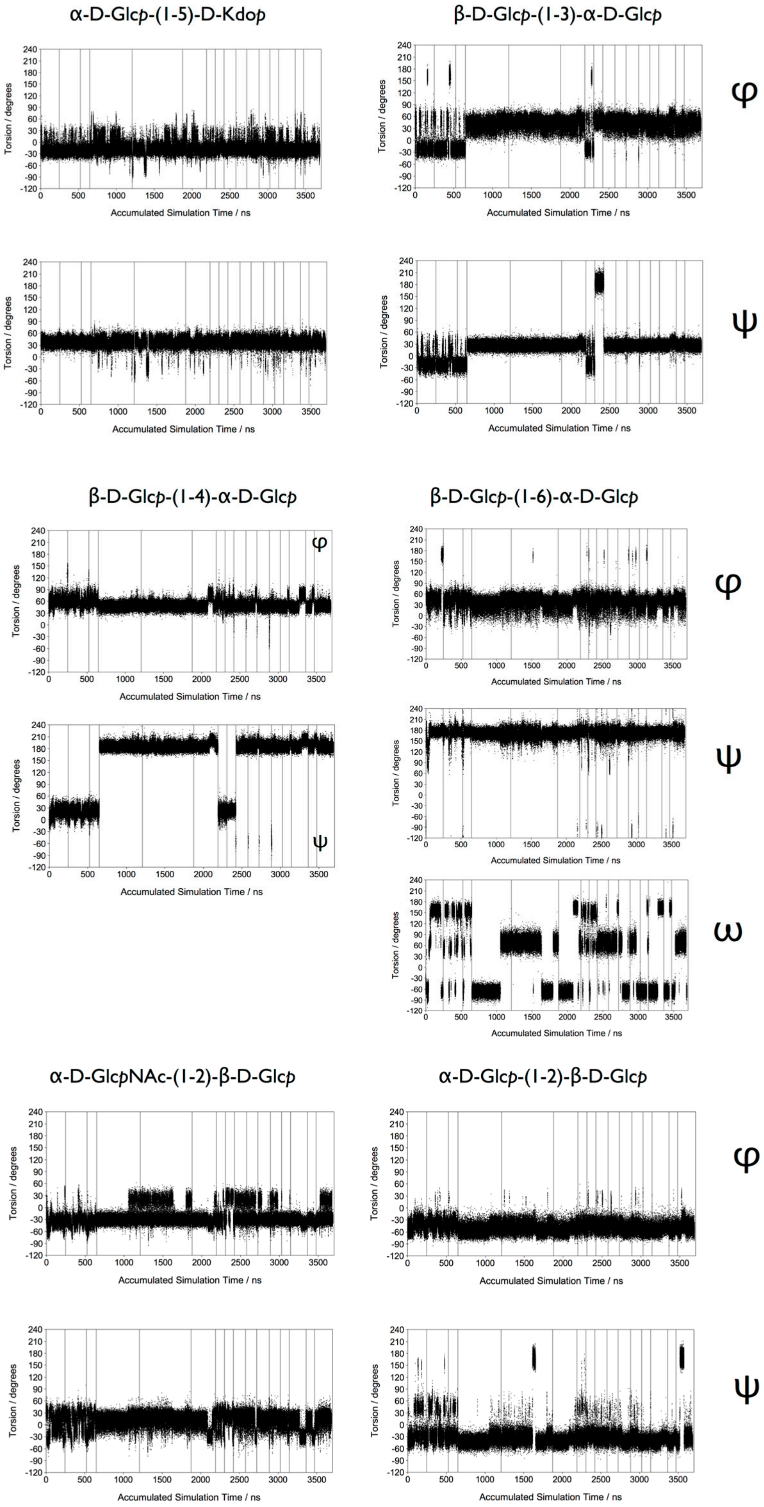

Figure S10. lgt2Δ OS: Dynamics of the glycosidic torsions on the microsecond time scale in explicit solvent at 310 K. Several trajectories were sampled using different starting structures. For efficient display the trajectories are shown as single continuous plots. The different MD runs are separated by a vertical line.

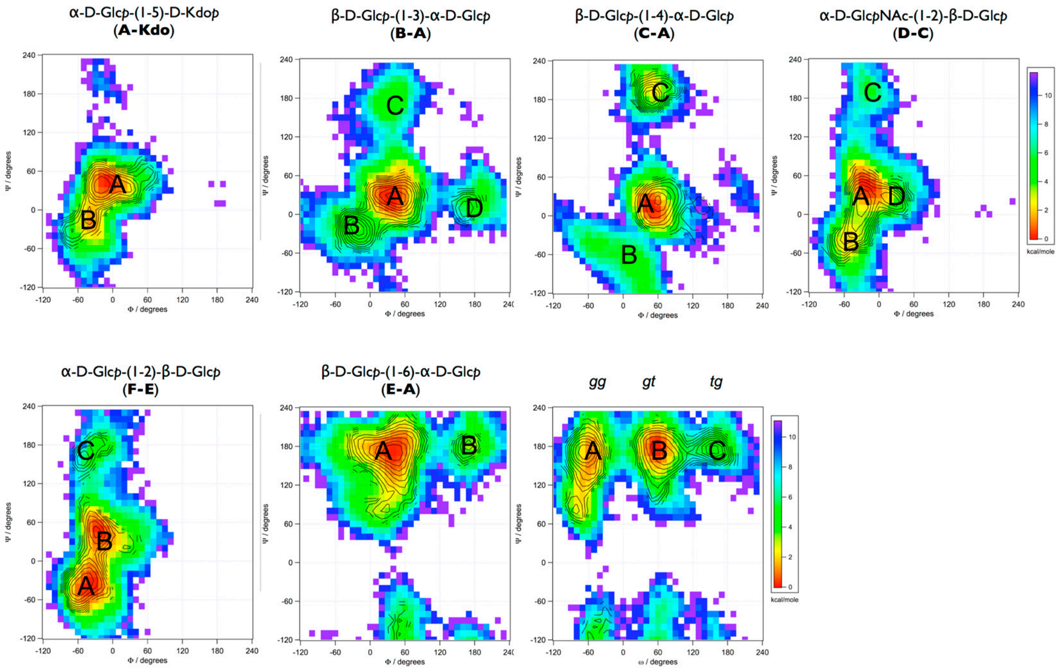

Figure S11. lgt2Δ OS: Local minima found for the glycosidic linkages from MD simulations in explicit solvent at 310 K (shown as contours). The data is calculated from several independent trajectories. It should be noted that the relative population between the local minima is artificial since no conformational equilibrium between the states has been sampled. The location of the minima in explicit solvent is very similar to their respective location in the free energy map (colored background) calculated based on HTMD simulation.
